# Reduced expression of microRNA-100 confers unfavorable prognosis in patients with bladder cancer

**DOI:** 10.1186/1746-1596-7-159

**Published:** 2012-11-22

**Authors:** Sheng Wang, Sheng Xue, Yuanqing Dai, Jianfu Yang, Zhijun Chen, Xiwu Fang, Wensheng Zhou, Wei Wu, Qingwen Li

**Affiliations:** 1 Department of Urology, the First Affiliated Hospital, Bengbu Medical College, Bengbu, 233030, People’s Republic of China; 2 Department of Geriatric Surgery, Xiangya Hospital, Central South University, Changsha, 410008, People’s Republic of China; 3 Department of Urology, the Third Xiangya Hospital of Central South University, Changsha, 410013, People’s Republic of China

**Keywords:** Bladder cancer, MicroRNA-100, Prognosis

## Abstract

**Objective:**

MicroRNA-100 (miR-100) has been demonstrated to be downregulated in bladder cancer tissues, and enforced expression of this miRNA may inhibit cell growth and colony formation of human bladder cancer 5637 cells in vitro. However, the clinical significance of miR-100 in human bladder cancer has not yet been elucidated. Thus, the aim of this study was to investigate the diagnostic and prognostic values of miR-100 in this disease.

**Methods:**

Expression levels of miR-100 in 126 pairs of bladder cancer and adjacent normal tissues were detected by TaqMan real-time quantitative RT-PCR assay. In order to determine its prognostic value, overall survival (OS) and progression-free survival (PFS) were evaluated using the Kaplan-Meier method, and multivariate analysis was performed using the Cox proportional hazard analysis.

**Results:**

Expression levels of miR-100 in bladder cancer tissues were significantly lower than those in adjacent normal tissues (mean expression level: 2.6 ± 1.2 vs. 3.9 ± 1.5, P < 0.001). When categorized into low vs. high expression, low miR-100 expression was negatively associated with the stage (P = 0.01), the recurrence (P = 0.008), the progression (P = 0.01), and the death (P < 0.001) of patients with bladder cancer. Moreover, low miR-100 expression clearly predicted poorer PFS (P = 0.001) and OS (P < 0.001). In the multivariate analysis, low miR-100 expression was an independent prognostic factor for both PFS (P = 0.01) and OS (P = 0.008).

**Conclusion:**

Our data offer the convincing evidence that miR-100 may play an important role in the progression of bladder cancer and that the reduced expression of this miRNA may be independently associated with shorter PFS and OS of patients, suggesting that miR-100 might be a potential marker for further risk stratification in the treatment of this cancer.

**Virtual slides:**

The virtual slides’ for this article can be found here: http://www.diagnosticpathology.diagnomx.eu/vs/1105483419841671

## Introduction

Bladder cancer is the seventh most common malignant neoplasm and the eighth leading cause of cancer death worldwide, with an estimated 68,810 new cases and 14,100 deaths in the USA in 2008 alone [1,2]. This malignancy affects the lining of the urinary bladder with a complicated, multifactorial etiology, involving both genetic and environmental factors. There are two principal forms of bladder cancers: low-grade superficial tumors and high-grade invasive cancer. The former are often papillary and multifocal, occasionally progress to invasive disease, and have a good prognosis, while the latter are usually nodular, metastasize during the early phase, and have a poor prognosis [3,4]. Approximately 70% of the patients who are diagnosed initially with superficial bladder cancer do not face a life-threatening situation; however, up to 70% of these patients developing at least one recurrence within 5 years [5]. Thus, the clinical outcome of bladder cancer patients is still poor in spite of the considerable progress made in the treatment of this disease. The challenges for controlling bladder cancer are the prevention of the recurrent disease and the inhibition of the disease progression during the treatment course. Clinicopathological parameters such as tumor grade and stage have been used clinically to evaluate pathologic events of bladder cancer; however, their sensitivity is relatively low [6]. Accumulating studies have found different marker expression in this cancer. For example, Bahadir et al. [7] indicated that CD10 expression may be strongly correlated with high tumor grade and stage in urothelial carcinoma of the bladder, and that CD10 may be associated with tumor progression in bladder cancer pathogenesis; Yildiz et al. [8] found that dual staining by p53 + CK20 cocktail may allow for histologic correlation and diminish the risk of losing the area of interest in limited biopsy specimens. Therefore, identification of novel effective molecular markers is of great significance for the improvement of diagnostic and prognostic techniques, and for the development of more efficient therapeutic strategies for patients with bladder cancer.

MicroRNAs (miRNAs), small non-coding, single strand RNA molecules that negatively regulate gene expression at the post-transcriptional level [9]. MiRNAs can downregulate gene expression by inducing the degradation or impairing the translation of target mRNAs [10]. A single miRNA may regulate hundreds of target mRNAs that are frequently grouped in a specific biological pathway. In the current estimate, about 900 unique miRNAs are encoded in the human genome, in part controlling the expression of more than a third of human genes [11]. Accumulating studies have demonstrated that miRNAs play important roles in angiogenic signaling, cell proliferation, apoptosis avoidance, and tumor invasion pathways [12]. In this context, miRNAs have been identified as promising alternative biomarkers for detecting cancer, informing prognosis, and monitoring treatment response. Recent studies have found that more than 40 miRNAs are involved in the tumorigenesis and tumor progression of urological cancers [13]. Many researchers have studied miRNA expression in bladder cancer by using various gene expression profiling approaches. Song et al. [14] in 2010 performed the miRNA microarray analysis with 25 cases of bladder cancers and adjacent normal bladder tissues. They identified a panel of 51 differentially expressed miRNAs with at least 2-fold differences in expression compared with the normal controls, including 20 up-regulated and 31 down-regulated miRNAs. In particular, miR-100 was found to be downregulated in bladder cancer. In another report of Oliveira et al. [15] in 2011, enforced expression of miR-100 may inhibit cell growth and colony formation of human bladder cancer 5637 cells in vitro. However, the clinical significance of miR-100 in human bladder cancer has not yet been elucidated. Thus, the aim of this study was to investigate the diagnostic and prognostic values of miR-100 in this disease.

## Materials and methods

### Patients and tissue samples

Our study was approved by the Ethics Committee of the First Affiliated Hospital of Bengbu Medical College, Xiangya Hospital, and the Third Xiangya Hospital. Informed consent was obtained from all of the patients.

One hundred and twenty-six pairs of primary bladder cancer and adjacent normal bladder tissues (≥ 3 cm away from bladder cancer tissues) were collected between 2007 and 2008 from the First Affiliated Hospital of Bengbu Medical College, Xiangya Hospital, and the Third Xiangya Hospital. Of these patients, 21 patients underwent radical cystectomy, 15 patients underwent partial cystectomy, and 90 patients underwent transurethral resection of bladder tumor (TURBT). After partial cystectomy and TURBT, Pirarubicin (THP) was used in intravesical therapy as weekly intravesical injection beginning within 24 hours after surgery. None of the patients had received preoperative treatment. All patients were classified according to the 1997 UICC TNM classification for the stage and OMS 2004 for the grade (LMP: low malignant potential; LG: low grade; HG: high grade). Tumor recurrence/progression was defined based on clinical, radiological, or histological diagnoses. The clinicopathologic characteristics of the patients with bladder cancer are shown in Table 1.

**Table 1:** **Clinicopathologic characteristics of 126 patients with bladder cancer**$\left(\overset{―}{X},\pm ,s\right)$

**Clinical features**	**N (%)**
**Mean age (minimum-maximum)**	66.8 (43–81)
**Sex**	
Male	87 (69.0)
Female	39 (31.0)
**Stage**	
Ta	48 (38.1)
T1	46 (36.5)
≥T2	32 (25.4)
**Grade**	
LMP	32 (25.4)
LG	43 (34.1)
HG	51 (40.5)
**Carcinoma in situ**	26 (20.6)
**Surgical procedure**	
TUR	90 (71.4)
Cystectomy	36 (28.6)
**Recurrence**	43 (34.1)
pTa	11 (25.6)
pT1	23 (53.5)
≥pT2	9 (20.9)
**Progression**	26 (18.8)
pTa	6 (23.1)
pT1	11 (42.3)
≥pT2	9 (34.6)
**Death**	65 (51.6)
pTa	12 (18.5)
pT1	30 (46.2)
≥pT2	23 (35.4)

TUR; transurethral resection; LMP: low malignant potential; LG: low grade; HG: high grade; Carcinoma in situ (CIS): 2 isolated CIS and 14 concomitant CIS.

To investigate the progression-free survival (PFS) or the overall survival (OS), we defined a time point of 36 months. PFS was defined as the time interval between initial surgical resection and the day of the appearance of new metastatic lesions. OS was determined from the date of surgery to the time of the last follow-up or cancer-related death. All patients who died from diseases other than bladder cancer or from unexpected events were excluded from the case collection.

### TaqMan real-time quantitative RT-PCR for miRNA

miR-100 expression in bladder cancer and adjacent normal bladder tissues was measured by real-time quantitative RT-PCR analysis. Briefly, total RNA was extracted from frozen samples using Trizol reagent (Invitrogen, Shanghai, China) according to the users’ instruction. RNA concentration and purity were measured using the NanoDrop ND-1000 spectrophotometer (NanoDrop Technologies, Houston, TX, USA). Only the samples with the OD A260/A280 ratio close to value of 2.0, which indicates that the RNA is pure, were subsequently analyzed. The miR-100 and RNU6B (as an internal control)-specific cDNA were synthesized from total RNA using gene-specific primers according to the TaqMan MicroRNA assays protocol (Applied Biosystems, Foster City, CA, USA). Each reaction included 1 × primer probe mix (TaqMan; ABI), 1× universal PCR master mix (TaqMan; ABI), and 200 ng of cDNA. Relative quantification of target miRNA expression was evaluated using the comparative cycle threshold (CT) method. Each sample was examined in triplicate and the raw data were presented as the relative quantity of target miRNA, normalized with respect to RNU6B.

### Statistical analysis

The software of SPSS version 13.0 for Windows (SPSS Inc, IL, USA) was used for statistical analysis. Data were expressed as means ± standard deviation (SD). Statistical analysis were performed with Fisher’s exact test for any 2 × 2 tables,Pearson *χ*^2^ test for non- 2 × 2 tables. The Cox proportional hazards model was used to evaluate the association of miR-100 expression with PFS and OS, respectively, after the operation. Survival curves were estimated based on the Kaplan-Meier method. Differences were considered statistically significant when *p* was less than 0.05.

## Results

### Reduced expression of miR-100 in bladder cancer

**Figure 1:**
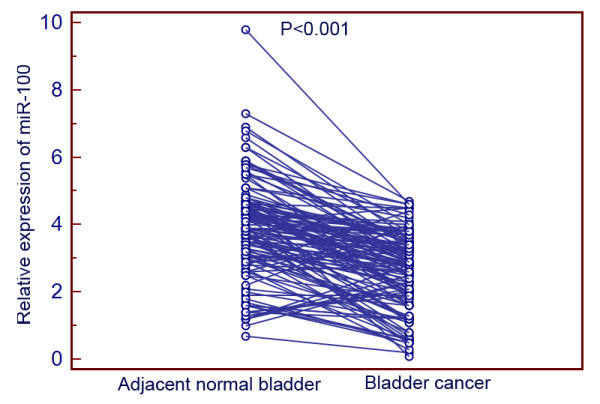
miR-100 expression in 126 pairs of bladder cancer and adjacent normal bladder tissues detected by quantitative real-time polymerase chain reaction (qRT-PCR) analysis.

To determine whether its expression differed between bladder cancer and adjacent normal bladder tissues, the expression levels of miR-100 were detected in 126 pairs of bladder cancer and adjacent normal bladder tissues normalized to RNU6B. As shown in Figure 1, the expression levels of miR-100 were found to be distinctly reduced in bladder cancer tissues compared to adjacent normal bladder tissues. The mean level of miR-100 expression in bladder cancer tissues was 2.6 ± 1.2, which was significantly lower on average than that in adjacent normal bladder tissues (3.9 ± 1.5, P < 0.001, Figure 1).

### Association between miR-100 expression and clinicopathological parameters of bladder cancer

Bladder cancer tissues expressing miR-100 at levels less than the median expression level (2.8) were assigned to the low expression group (mean expression value 1.8, n = 78), and those samples with expression above the median value were assigned to the high expression group (mean expression value 3.9, n = 48). The miR-100 expression was associated with several clinicopathological parameters as shown in Table 2. The bladder cancer patients with low miR-100 expression more frequently had the high tumor stage (P = 0.01), the tumor recurrence (P =0.008), the advanced tumor progression (P = 0.01), and the death (P < 0.001) of patients with bladder cancer.

**Table 2:** Association between miR-100 expression and clinicopathological parameters of bladder cancer

**Clinical features**	**N**	**miR-100**		***P***
		**Low (n = 78)**	**High (n = 48)**	
**Sex**				
Male	87	55 (63.2)	32 (36.8)	0.1
Female	39	23 (59.0)	16 (40.9)	
**Age (years)**				
≤70	55	30 (54.5)	25 (45.5)	0.08
>70	71	48 (67.6)	23 (32.4)	
**Stage**				
Ta	48	20 (41.7)	28 (58.3)	**0.01**
T1	46	30(65.2)	16 (34.8)	
≥T2	32	28 (87.5)	4 (12.5)	
**Grade**				
LMP	32	20 (62.5)	12 (37.5)	0.2
LG	43	26(60.5)	17(39.5)	
HG	51	32(62.7)	19(37.3)	
**Carcinoma in situ**				
Yes	26	18 (69.2)	8(30.8)	0.09
No	100	60 (60.0)	40 (40.0)	
**Recurrence**				
Yes	43	36(83.7)	7 (16.3)	**0.008**
No	73	42 (57.5)	31(42.5)	
**Progression**				
Yes	26	20 (76.9)	6 (23.1)	**0.01**
No	100	58 (58.0)	42(42.0)	
**Death**				
Yes	65	62 (95.4)	3 (4.6)	**< 0.001**
No	61	16 (26.2)	45 (73.8)	

TUR; transurethral resection; LMP: low malignant potential; LG: low grade; HG: high grade.

### Univariate analysis of the correlation between miR-100 and the survival of patients with bladder cancer

**Figure 2:**
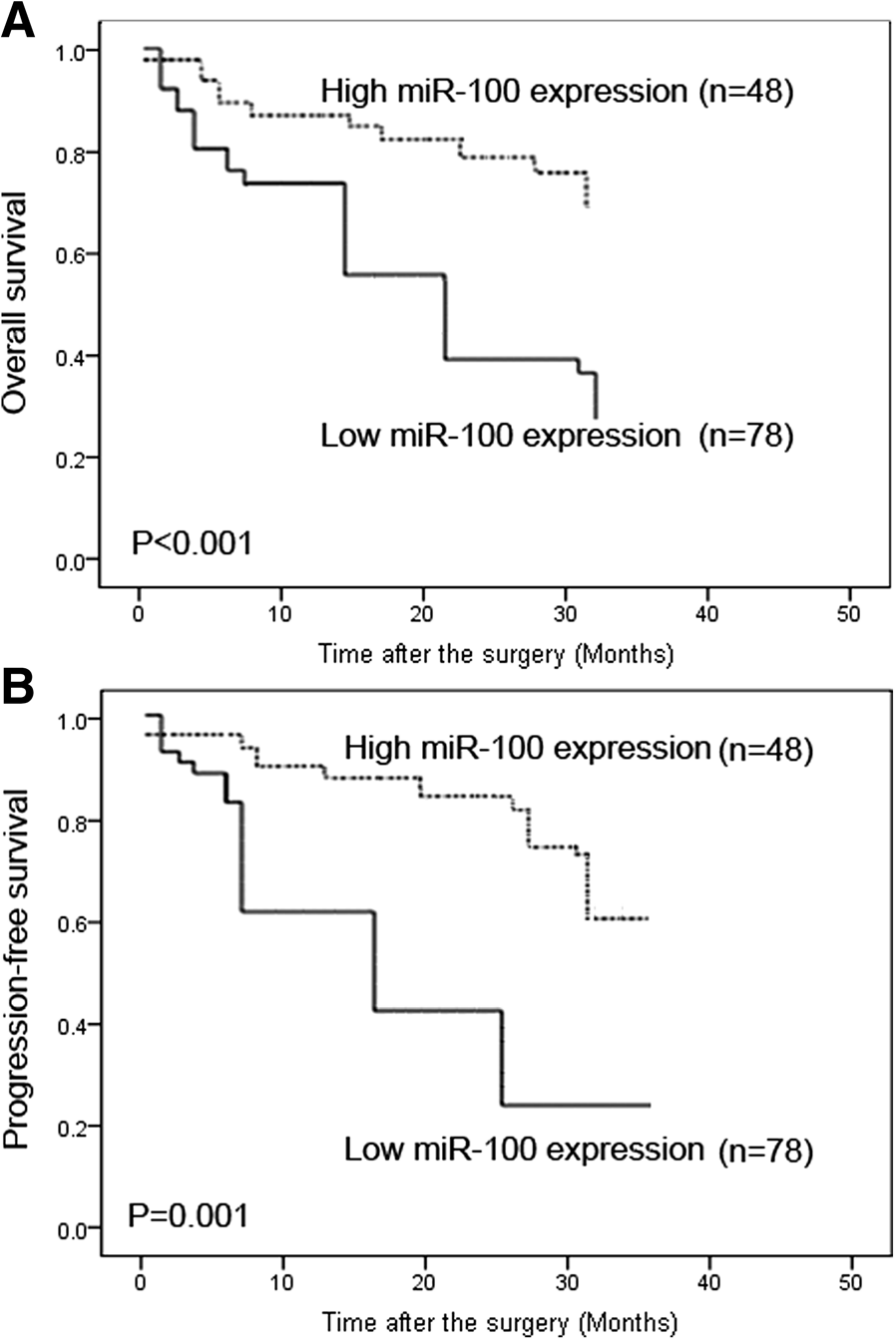
**Kaplan-Meier survival curves for bladder cancer patients according to the expression of miR-100.** (**A**) Progression-free survival (PFS); (**B**) overall survival (OS).

To investigate OS or PFS, we defined a time point of 36 months. During the time study, 26 (20.6%) patients presented a progression and 100 (79.4%) did not. In patients who progressed, 20 (76.9%) had low miR-100 expression. The 3-year OS rates of patients who were low (n = 78) and high (n = 48) for miR-100 expression were 29.5% and 68.8%, respectively (Table 3). In univariate analysis, low miR-100 expression (P < 0.001), the stage (P = 0.006), the grade (P = 0.02), and CIS (P = 0.01) were significant predictors of short OS (Table 3). For the 3-year PFS rates, patients with low miR-100 expression represented 20.5% and patients without, 54.2% (Table 3). Low miR-100 expression (P = 0.001), stage (P = 0.008), grade (P = 0.03), and CIS (P = 0.02) were also negative predictors of the PFS (Table 3). Figure 2 shows the OS and PFS curves with respect of miR-100 expression. More importantly, the prognostic value of miR-100 in bladder cancer patients was higher than that of tumor stage (P value for OS: <0.001 vs. 0.006; for PFS: 0.001 vs. 0.008; Table 3), grade (P value for OS: <0.001 vs. 0.03; for PFS: 0.001 vs. 0.02; Table 3) and CIS (P value for OS: <0.001 vs. 0.02; for PFS: 0.001 vs. 0.01; Table 3).

**Table 3:** Univariate analyses of various clinicopathological parameters in relation to survival of patients with bladder cancer

**Clinical features**	**N**	**OS**		**PFS**	
		**3-year survival (%)**	***P***	**3-year survival (%)**	***P***
**Sex**					
Male	87	34 (39.1)	0.08	24 (27.6)	0.08
Female	39	22 (56.4)		18 (46.2)	
**Age (years)**					
≤70	55	20 (36.4)	0.1	13 (23.6)	0.1
>70	71	36 (50.7)		29 (40.8)	
**Stage**					
Ta	48	30 (62.5)	0.006	26 (54.2)	0.008
T1	46	22 (47.8)		12 (26.1)	
≥T2	32	4 (12.5)		4 (12.5)	
**Grade**					
LMP	32	18 (56.2)	0.02	17 (53.1)	0.03
LG	43	21 (48.8)		15 (34.9)	
HG	51	17 (33.3)		10 (19.6)	
**Carcinoma in situ**					
Yes	26	16 (61.5)	0.01	12 (46.2)	0.02
No	100	40 (40.0)		30 (30.0)	
**miR-100**					
High	48	33 (68.8)	<0.001	26 (54.2)	0.001
Low	78	23 (29.5)		16 (20.5)	

### Multivariate analysis of prognostic variables in patients with bladder cancer

In multivariate OS analysis, miR-100, stage, grade, and CIS was entered into the Cox proportional hazard analysis. miR-100 expression (OR, 10.1; 95% CI, 1.5 ~ 27.2; P = 0.008), stage (OR, 7.6, 95% CI, 1.2 ~ 22.3; P = 0.01), grade (OR, 3.9, 95% CI, 1.0 ~ 10.2; P = 0.04) and CIS (OR, 4.8, 95% CI, 1.0 ~ 12.5; P = 0.03) retained significance as a prognostic factor of a short OS (Table 4). In multivariate PFS analysis, miR-100 expression (OR, 8.3; 95% CI, 1.3 ~ 23.7; P = 0.01), stage (OR, 6.5, 95% CI, 1.0 ~ 13.1; P = 0.02), grade (OR, 1.6, 95% CI, 0.2 ~ 3.7; P = 0.08) and CIS (OR, 3.1, 95% CI, 1.0 ~ 9.6; P = 0.04) were independently significant prognostic factors (Table 4).

**Table 4:** Multivariate analyses with the Cox log-rank test of the effect on OS and PFS

	**OS**		**PFS**	
	**Hazard ratio (95% CI)**	***P***	**Hazard ratio (95% CI)**	***P***
**Stage**	7.6 (1.2 ~ 22.3)	**0.01**	6.5 (1.0 ~ 13.1)	**0.02**
**Grade**	3.9 (1.0 ~ 10.2)	**0.04**	1.6 (0.2 ~ 3.7)	0.08
**Carcinoma in situ**	4.8 (1.0 ~ 12.5)	**0.03**	3.1 (1.0 ~ 9.6)	**0.04**
**miR-100**	10.1 (1.5 ~ 27.2)	**0.008**	8.3 (1.3 ~ 23.7)	**0.01**

## Discussion

In our retrospective exploratory study, there are four main findings as following: first, the expression levels of miR-100 in bladder cancer tissues were significantly lower than those in adjacent normal tissues; next, low miR-100 expression was associated with advanced tumor progression in bladder cancer; then, low miR-100 expression was associated with shorter PFS and OS in bladder cancer; and finally, miR-100 was identified as an independent prognostic marker for bladder cancer. To our best of knowledge, this is the first study on the clinical significance of miR-100 expression using a large number of bladder cancer cases.

MiR-100, together with miR-99a and miR-99b, belongs to the miR-100 family. It is the oldest known animal miRNA and is extensively expressed in vertebrates [16]. Recent studies have demonstrated that the aberrant expression of miR-100 is associated with tumorigenesis and tumor progression. Notably, the roles of miR-100 in different cancers are quite contradictory as it can behave either as an oncogene or a tumor suppressor gene, depending on the tumor type examined. MiR-100 is downregulated in nasopharyngeal cancer, oral squamous cell carcinoma, ovarian cancer, hepatocellular carcinoma, hepatoblastoma and bladder cancer, whereas its upregulation has been described in acute myeloid leukemia, medulloblastomas, gastric cancer, pancreatic cancer and prostate cancer [17-27]. Especially in bladder cancer, both the previous studies of Oliveira et al. [15] and Song et al. [14] found the decreased expression of miR-100 in bladder cancer cells. In line with these findings, our data also validated the downregulation of miR-100 in a large number of clinical bladder cancer cases. Functionally, the aberrant expression of miR-100 in acute myeloid leukemia cells can promote cell proliferation and block granulocyte/monocyte differentiation [22]. Oral squamous cell carcinoma cells transfected with miR-100 may modify the expression of a number of oncogenes including ID1, EGR2, MMP13, and FGFR3 [18]. Henson et al. [28] also demonstrated that miR-100 could be used therapeutically to increase the sensitivity of oral squamous cell carcinoma cells to ionizing radiation by decreasing the expression of the genes that confer radioresistance. These findings raise the possibility that miR-100 might have an important role in the development or pathogenesis of various cancers. Similarly, our data in the present study showed that the downregulation of miR-100 may more frequently occur in bladder cancer tissues with aggressive clinicopathological features and poor prognosis.

## Conclusion

In conclusion, our data offer the convincing evidence that miR-100 may play an important role in the progression of bladder cancer and that the reduced expression of this miRNA may be independently associated with shorter PFS and OS of patients, suggesting that miR-100 might be a potential marker for further risk stratification in the treatment of this cancer. However, the mechanism by which miR-100 was downregulated in bladder cancer is still unclear. This study is hypothesis generating, and that further prospective analysis should be worth doing.

## Competing interests

The authors’ declare that they have no competing interests.

## Authors’ contributions

SW and QL: wrote the manuscript, carried out study design and experiments; SX, YD, JY, ZC, and XF: collected the samples; WZ: carried out part of experiments; WW: wrote the manuscript and designed the study. All authors read and approved the final manuscript.
